# MetaGS: an accurate method to impute and combine SNP effects across populations using summary statistics

**DOI:** 10.1186/s12711-022-00725-7

**Published:** 2022-06-02

**Authors:** Abdulqader Jighly, Haifa Benhajali, Zengting Liu, Mike E. Goddard

**Affiliations:** 1 Agriculture Victoria, AgriBio, Centre for AgriBiosciences, Bundoora, VIC 3083 Australia; 2 Department of Animal Breeding and Genetics, Interbull Centre, Swedish University of Agricultural Sciences, Box 7023, 750 07 Uppsala, Sweden; 3 IT Solutions for Animal Production (vit), Heinrich-Schroeder-Weg 1, 27283 Verden, Germany; 4 Faculty of Veterinary and Agricultural Science, University of Melbourne, Parkville, VIC 3010 Australia

## Abstract

**Background:**

Meta-analysis describes a category of statistical methods that aim at combining the results of multiple studies to increase statistical power by exploiting summary statistics. Different industries that use genomic prediction do not share their raw data due to logistic or privacy restrictions, which can limit the size of their reference populations and creates a need for a practical meta-analysis method.

**Results:**

We developed a meta-analysis, named MetaGS, that duplicates the results of multi-trait best linear unbiased prediction (mBLUP) analysis without accessing raw data. MetaGS exploits the correlations among different populations to produce more accurate population-specific single nucleotide polymorphism (SNP) effects. The method improves SNP effect estimations for a given population depending on its relations to other populations. MetaGS was tested on milk, fat and protein yield data of Australian Holstein and Jersey cattle and it generated very similar genomic estimated breeding values to those produced using the mBLUP method for all traits in both breeds. One of the major difficulties when combining SNP effects across populations is the use of different variants for the populations, which limits the applications of meta-analysis in practice. We solved this issue by developing a method to impute missing summary statistics without using raw data. Our results showed that imputing summary statistics can be done with high accuracy (*r* > 0.9) even when more than 70% of the SNPs were missing with a minimal effect on prediction accuracy.

**Conclusions:**

We demonstrated that MetaGS can replace the mBLUP model when raw data cannot be shared, which can lead to more flexible collaborations compared to the single-trait BLUP model.

## Background

The simultaneous estimation of the genomic effect on quantitative traits using all available variants, named genomic prediction [1], has revolutionized the fields of plant breeding [2], animal breeding [3] and personalized medicine [4]. This is because it considers the linkage disequilibrium (LD) between variants when they are all fitted in a single model instead of attributing the same variation to multiple variants in high LD when running single variant association analyses [5]. Complex traits are usually controlled by a large number of genes with small effects, which requires a very large population to train the prediction equation with any reliability [6]. Such large reference sets with genotyped and phenotyped individuals may not be available for all populations and/or traits which requires combining different populations in a single analysis. Ideally, a joint analysis using the raw phenotypic and genotypic data of different populations can be run to get more accurate predictions. However, restrictions on sharing raw data and privacy regulations limit such possibilities [7]. While sharing individual-level data is generally constrained, it is widely acceptable to share summary statistics that are descriptive for the prediction outcome and cannot be used to infer the original data. Such summary statistics can be combined in a data analysis frame called meta-analysis [8].

A meta-analysis refers to the inference of more robust outcomes by combining the results of multiple studies in a single analysis, in other words, the analysis of analyses. The term “meta-analysis” was first proposed by Glass [9]. Multi-trait across country evaluation (MACE) is a traditional example of a meta-analysis in animal breeding [10], which uses as input the estimated breeding values (EBV) of bulls calculated within each country rather than the bull’s raw data. VanRaden and Sullivan [11] extended the MACE model to include genomic information (GMACE) which facilitated better prediction for young bulls with no daughter proofs [12]. GMACE has been successfully used for over a decade and it has a proven capability to increase the reliability of bull’s genomic EBV (GEBV). However, an alternative approach is required to analyze data from other organisms.

To estimate the substitution effect of single nucleotide polymorphisms (SNPs) when raw data are not available, different meta-based models were proposed regardless of whether the variants were fitted independently as in genome-wide association studies (GWAS) or jointly as in genomic prediction studies [13–16]. Although these models resulted in higher prediction accuracy compared to all participant individual data, most of them showed a lower accuracy when compared to the joint analysis that uses individual-level data, as they depend on approximation [14]. The model proposed by Vandenplas et al. [16] was designed to exploit both raw data as well as summary statistics in the same analysis by fitting multiple records per trait and it showed minimal approximation compared to other models using simulated data. However, it assumes that the genetic correlation among all participant populations is equal to 1 which may not be the case for many populations/traits. Another drawback with these models is that they all require participant populations to be genotyped with the same set of SNPs, which limit their practical application in real life.

The objective of the present study was to develop a meta-analysis (named MetaGS) that mimics the multiple-trait best linear unbiased prediction (mBLUP) model using raw data with limited approximation. Our model assumes that each population would benefit from its correlation with other populations to gain more accurate population-specific SNP effects. Participant data holders are expected to share the LD matrices, the frequencies, and the effects of their SNPs as well as the variance of the direct genomic value and error variance in their populations. The model is sufficiently flexible to allow for imputing missing variants and their effects in different populations using the previous summary statistics without accessing the individual-based data. The model was tested on Australian Holstein and Jersey cattle populations for milk, fat and protein yields and it showed that it can duplicate the joint analysis that uses the raw data with no approximation.

## Methods

Our multi-trait BLUP model assumes that the effects of a SNP in population $i$ and $j$ (${\mathbf{g}}_{i}$ and ${\mathbf{g}}_{j}$) are genetically correlated with the same correlation as the genetic correlation between true breeding values in different populations. Such a correlation can be inferred from other tests such as the correlation between estimated breeding values or the MACE. Within population $i$ ($i$ = 1,…,*c*), the SNP effects are ${\mathbf{g}}_{i}$, where ${\mathbf{g}}_{i}$ is a vector of SNP effects in population $i$.

### SNP effect estimation in a single population

The input to the meta-analysis are SNP effects estimated within each population excluding foreign data. We assume that the input individual estimates of SNP effects for population $i$ are estimated with a SNP BLUP model [17] that would be equivalent to:

1 $${\mathbf{y}}_{i} = \mu_{i} {\mathbf{1}} + {\mathbf{Z}}_{i} {\mathbf{g}}_{i} + {\mathbf{e}}_{i} ,$$

where ${\mathbf{y}}_{i}$ is a vector of phenotypes (in our data, average of daughter phenotypes) of the training or reference population corrected for all effects except the additive genetic effects explained by the SNPs; $\mu_{i}$ is the general mean of population $i$; **1** is a vector of 1s; ${\mathbf{Z}}_{i}$ represents the design matrix for the genotypes of reference individuals. Genotypic values of reference individuals take three possible values: $2 - 2p_{ij}$, $1 - 2p_{ij}$ and $0 - 2p_{ij}$ for genotypes *AA*, *AB* or *BB*, respectively [18], $p_{ij}$ is the allele frequency of SNP $j$ ($j$ = 1, …, $m$) of population $i$; ${\mathbf{e}}_{i}$ is a vector of residual effects for the reference population (e.g. in a sire genomic model) with a (co)variance matrix as follows:

2 $$\left[ {var({\mathbf{e}}_{i} )} \right]^{ - 1} = {\mathbf{R}}_{i}^{ - 1} = diag\left\{ {n_{ik} \sigma_{{e_{i} }}^{ - 2} } \right\},$$

where $\sigma_{{e_{i} }}^{2}$ is the error variance of population $i$, and $n_{ik}$ is the effective number of daughters contributing to ${\mathbf{y}}_{ik}$ of the reference individual $k$ in population $i$. We tested the use of population-specific allele frequencies (which vary from  one population to another) or a unified frequency (using pooled individuals from all populations) and we did not get any difference in the results (results not shown). Strandén and Christensen [19] showed that SNP-BLUP is invariant to the frequencies used for genotype centering if the model includes a mean which agrees with our results.

Under the SNP BLUP model [17], SNP effects are distributed as:

3 $$var({\mathbf{g}}_{i} ) = {\mathbf{B}}_{i} \sigma_{i}^{2} ,$$

4 $${\text{where}}\;{\mathbf{B}}_{i} = \frac{1}{{\mathop \sum \nolimits_{j} 2p_{ij} \left( {1 - p_{ij} } \right)}}{\mathbf{I}} = \theta_{i} {\mathbf{I}},$$

with $\sigma_{i}^{2}$ being the variance of the direct genomic values (DGV) of population $i$.

DGV represents the sum of all SNP effects:

5 $${\text{DGV}}_{ik} = {\mathbf{z}}_{ik} {\mathbf{g}}_{i} ,$$

where ${\text{DGV}}_{ik}$ is the breeding value of individual $k$ explained by SNPs; ${\mathbf{z}}_{ik}$ is a row in the design matrix ${\mathbf{Z}}_{i}$ corresponding to individual $k$. For this model, the mixed model equations for population $i$ are:

6 $$\left[ {\begin{array}{*{20}c} {{\mathbf{1^{\prime}}}{\mathbf{R}}_{i}^{ - 1} {\mathbf{1}}} & {{\mathbf{1^{\prime}}}{\mathbf{R}}_{i}^{ - 1} {\mathbf{Z}}_{i} } \\ {{{\mathbf{Z}}_{i}}^{\prime } {\mathbf{R}}_{i}^{ - 1} {\mathbf{1}}} & {{{\mathbf{Z}}_{i}}^{\prime } {\mathbf{R}}_{i}^{ - 1} {\mathbf{Z}}_{i} + \sigma_{i}^{ - 2} {\mathbf{B}}_{i}^{ - 1} } \\ \end{array} } \right]\left[ {\begin{array}{*{20}c} {\hat{\mu }_{i} } \\ {{\hat{\mathbf{g}}}_{i} } \\ \end{array} } \right] = \left[ {\begin{array}{*{20}c} {{\mathbf{1^{\prime}}}{\mathbf{R}}_{i}^{ - 1} {\mathbf{y}}_{i} } \\ {{{\mathbf{Z}}_{i}}^{\prime } {\mathbf{R}}_{i}^{ - 1} {\mathbf{y}}_{i} } \\ \end{array} } \right].$$

### (Co)variance of SNP effects in different populations

For the multi-trait BLUP model, SNP effects from different populations have the following (co)variance matrix:

7 $$var\left[ {\begin{array}{*{20}c} {{\mathbf{g}}_{1} } \\ {{\mathbf{g}}_{2} } \\ \vdots \\ {{\mathbf{g}}_{c} } \\ \end{array} } \right] = \left[ {\begin{array}{*{20}c} {\sigma_{1}^{2} {\mathbf{B}}_{1} } & {\sigma_{12} {\mathbf{B}}_{12} } & \cdots & {\sigma_{1c} {\mathbf{B}}_{1c} } \\ {} & {\sigma_{2}^{2} {\mathbf{B}}_{2} } & \cdots & {\sigma_{2c} {\mathbf{B}}_{2c} } \\ {} & {} & \ddots & \vdots \\ {symm.} & {} & {} & {\sigma_{c}^{2} {\mathbf{B}}_{c} } \\ \end{array} } \right] = {\mathbf{G}},$$

where $\sigma_{1c}$ is the DGV covariance between population 1 and $c$.

Similar to the definition of matrix ${\mathbf{B}}_{i}$ for population $i$, matrix ${\mathbf{B}}_{i,c}$ for the two populations relies on the assumption that the same set of SNPs is used in the two populations:

8 $${\mathbf{B}}_{i,c} = \frac{1}{{\sqrt {\mathop \sum \nolimits_{j} 2p_{ij} \left( {1 - p_{ij} } \right)} \sqrt {\mathop \sum \nolimits_{j} 2p_{cj} \left( {1 - p_{cj} } \right)} }}{\mathbf{I}} = \sqrt {\theta_{i} \theta_{c} } {\mathbf{I}}.$$

The (co)variance matrix of the population SNP effects, Eq. (7), becomes:

9 $${\mathbf{G}} = var\left[ {\begin{array}{*{20}c} {{\mathbf{g}}_{1} } \\ {{\mathbf{g}}_{2} } \\ \vdots \\ {{\mathbf{g}}_{c} } \\ \end{array} } \right] = \left[ {\begin{array}{*{20}c} {\sigma_{1}^{2} \theta_{1} {\mathbf{I}}} & {\sigma_{12} \sqrt {\theta_{1} \theta_{2} } {\mathbf{I}}} & \cdots & {\sigma_{1c} \sqrt {\theta_{1} \theta_{c} } {\mathbf{I}}} \\ {} & {\sigma_{2}^{2} \theta_{2} {\mathbf{I}}} & \cdots & {\sigma_{2c} \sqrt {\theta_{2} \theta_{c} } {\mathbf{I}}} \\ {} & {} & \ddots & \vdots \\ {symm.} & {} & {} & {\sigma_{c} \theta_{c} {\mathbf{I}}} \\ \end{array} } \right],$$

and its inverse matrix is:

10 $${\mathbf{G}}^{ - 1} = \left[ {\begin{array}{*{20}c} {{\mathbf{G}}^{11} } & {{\mathbf{G}}^{12} } & \cdots & {{\mathbf{G}}^{1c} } \\ {} & {{\mathbf{G}}^{22} } & \cdots & {{\mathbf{G}}^{2c} } \\ {} & {} & \ddots & \vdots \\ {symm.} & {} & {} & {{\mathbf{G}}^{cc} } \\ \end{array} } \right].$$

### Estimation of SNP effects in the meta-analysis model (MetaGS)

The effects of the meta-analysis model are estimated using the following mixed model equations:

11 $$\left[ {\begin{array}{*{20}c} {{\mathbf{Z}}_{1}^{\prime } {\mathbf{R}}_{1}^{ - 1} {\mathbf{Z}}_{1} + {\mathbf{G}}^{11} } & \cdots & {{\mathbf{G}}^{1c} } \\ {} & \ddots & \vdots \\ {symm.} & {} & {{\mathbf{Z}}_{c}^{\prime } {\mathbf{R}}_{c}^{ - 1} {\mathbf{Z}}_{c} + {\mathbf{G}}^{{{\text{cc}}}} } \\ \end{array} } \right] \times \left[ {\begin{array}{*{20}c} {{\hat{\mathbf{g}}}_{1} } \\ \vdots \\ {{\hat{\mathbf{g}}}_{c} } \\ \end{array} } \right] = \left[ {\begin{array}{*{20}c} {{\mathbf{Z}}_{1}^{\prime } {\mathbf{R}}_{1}^{ - 1} {\mathbf{y}}_{1} } \\ \vdots \\ {{\mathbf{Z}}_{c}^{\prime } {\mathbf{R}}_{c}^{ - 1} {\mathbf{y}}_{c} } \\ \end{array} } \right].$$

All the terms in Eq. (11) can be derived from the individual population analyses. Data holders need to submit the components that allow to build Eq. (11) which are for population $i$: (1) SNP effect estimates ${\mathbf{g}}_{i}$ or the right-hand side (RHS) ${\mathbf{Z}}_{i}^{\prime } {\mathbf{R}}_{i}^{ - 1} {\mathbf{y}}_{i}$; (2) ${{\mathbf{Z}}_{i}}^{\prime } {\mathbf{R}}_{i}^{ - 1} {\mathbf{Z}}_{i}$ for a measure of prediction error (co)variances of the SNP effect estimates; (3) marker allele frequencies of a reference SNP allele such as allele *A* in the population; and (4) the variance of direct genomic values. All the participating data holders must code the two SNP alleles *A* and *B* in the same way (i.e. using a specific reference genome, homozygosity for the reference allele can be coded as 0, homozygosity for the alternative allele can be coded as 2, and heterozygosity can be coded as 1), so they end with equivalent ${\mathbf{g}}_{i}$ estimations and ${{\mathbf{Z}}_{i}}^{\prime } {\mathbf{R}}_{i}^{ - 1} {\mathbf{Z}}_{i}$ matrices across populations.

In the present paper, we assumed that there was no residual polygenic (RPG) effect. However, if an RPG effect exists, it can be fitted during the estimation of SNP effects for each individual population following Liu et al. [17] without affecting the meta-analysis equations. This was validated using commercial Brown Swiss bulls’ data from six countries, but the (results not shown here).

It is not necessary for data holders to submit multiple ${{\mathbf{Z}}_{i}}^{\prime } {\mathbf{R}}_{i}^{ - 1} {\mathbf{Z}}_{i}$ matrices if they phenotyped different sets of individuals for different traits (assuming similar reliabilities across traits) as these matrices are supposed to be correlated. Instead, they are required to submit the number of reference individuals ($\alpha$) used to generate ${{\mathbf{Z}}_{i}}^{\prime } {\mathbf{R}}_{i}^{ - 1} {\mathbf{Z}}_{i}$ as well as the number of phenotyped reference individuals (bulls in our case) for trait $i$ ($n_{i}$). The ${{\mathbf{Z}}_{i}}^{\prime } {\mathbf{R}}_{i}^{ - 1} {\mathbf{Z}}_{i}$ matrix can be rescaled with the number of phenotypes to avoid overestimating the magnitude of populations with missing phenotypes using the following equation:

12 $${\text{rescaled}}\;{{\mathbf{Z}}_{i}}^{\prime } {\mathbf{R}}_{i}^{ - 1} {\mathbf{Z}}_{i} = n_{i} /\alpha { } \times {{\mathbf{Z}}_{i}}^{\prime } {\mathbf{R}}_{{\text{i}}}^{ - 1} {\mathbf{Z}}_{i} .$$

If reliabilities across traits were different, data holders should submit multiple ${{\mathbf{Z}}_{i}}^{\prime } {\mathbf{R}}_{i}^{ - 1} {\mathbf{Z}}_{i}$ matrices.

### Handling different sets of SNPs between populations

Here, we propose a method to account for different SNP datasets used by different populations. In this method, we expand the list of SNPs to include all SNPs used by any of the participating populations. Equation (6) shows how the right-hand sides (RHS) of the mixed model equations for each population can be obtained from the left-hand sides (LHS) that the population provides i.e. the design matrix and the SNP solutions. However, these RHS are missing for SNPs that are not used by that population, so we impute the missing RHS as follows. We assume that, due to LD among the SNPs, the genotypes for the complete set of SNPs (${\mathbf{Z}}_{c}$) on the bulls used by population $i$ are related to the genotypes for non-missing SNPs (${\mathbf{Z}}_{i}$) by ${\mathbf{Z}}_{c} = {\mathbf{Z}}_{i} {\mathbf{T}}$, where ${\mathbf{T}}$ is an $i \times c$ matrix with $i$ the number of SNPs used by population $i$ and $c$ the total number of SNPs.

The previous assumption requires a set of animals with both the missing and non-missing SNPs recorded. This could be done within population $i$ or by using a reference set of animals. ${\mathbf{T}}$ can be calculated on the reference population by:

13 $${\mathbf{T}} = \left( {{\mathbf{Z}}_{i}^{\prime } {\mathbf{Z}}_{i} } \right)^{{ - {\mathbf{1}}}} {\mathbf{Z}}_{i}^{\prime } {\mathbf{Z}}_{c} .$$

Then, the RHS for the missing SNPs can be calculated by:

14 $${\mathbf{Z}}_{c}^{\prime } {\mathbf{R}}_{i}^{ - 1} {\mathbf{y}} = {\mathbf{T^{\prime}Z}}_{i}^{\prime } {\mathbf{R}}_{i}^{ - 1} {\mathbf{y}},$$

and the LHS by:

15 $${\mathbf{Z}}_{c}^{\prime } {\mathbf{R}}_{i}^{ - 1} {\mathbf{Z}}_{c} = {\mathbf{T^{\prime}Z}}_{i}^{\prime } {\mathbf{R}}_{i}^{ - 1} {\mathbf{Z}}_{i} {\mathbf{T}}.$$

This gives all the necessary inputs for Eq. (11). Therefore, the method does not directly impute the input SNPs but instead imputes the underlying matrices that are adequate to estimate SNP effects using the MetaGS model based on the LD between existing and missing SNPs.

After the complete equations have been solved and yield prediction equations for each population based on the complete SNP set, the solutions for the original SNP set of population $i$ can be obtained by:

16 $${\mathbf{g}}_{i} = {\mathbf{Tg}}_{c} .$$

where ${\mathbf{g}}_{c}$ are the SNP solutions for population $i$ based on the complete SNP set and ${\mathbf{g}}_{i}$ are the solutions for the original SNP set of population $i$. In the validation analysis of this paper, we calculated multiple submatrices for ${\mathbf{T}}$ for every 200 adjacent SNPs on the same chromosome to make the matrix invertible. Therefore, the full ${\mathbf{T}}$ matrix became a block-diagonal matrix.

### Validation data

The data involved Australian Holstein (4627 bulls) and Jersey (1178 bulls) populations genotyped with the 50k SNP chip. After filtering for minor allele frequency, the dataset included 40,850 SNPs. Three traits were considered, milk yield, milk fat and milk protein yields. The reference population included 1071 Jersey and 4105 Holstein bulls born before 2010. The validation set contained 107 Jersey and 522 Holstein bulls born after 2010. The raw data were analysed using MTG2 [20] for comparison with the new model (MetaGS) considering a univariate model for each breed independently [single trait (ST)], as well as a bivariate, multi-breed model (mBLUP). The bivariate model assumes that the phenotypes of the two breeds for the same trait are two different correlated traits. The genetic correlation between Holstein and Jersey bulls was estimated using MTG2 and fitted in the meta-analysis to ensure that the same ${\mathbf{G}}$ matrix was built with both models. Correlations were 0.54, 0.36 and 0.33 for milk, fat and protein yields, respectively. The prediction accuracy was inferred from correlations between DGV and the phenotypes of the validation population.

To test the accuracy of rescaling the ${\mathbf{Z^{\prime}R}}^{{ - {\mathbf{1}}}} {\mathbf{Z}}$ matrix, Eq. (12) was applied after masking different proportions (ranging from 0.01 to 0.95 with a 0.01 increment) of the Holstein and Jersey populations. The accuracy was inferred from the correlation between the rescaled ${\mathbf{Z^{\prime}R}}^{{ - {\mathbf{1}}}} {\mathbf{Z}}$ matrices and the original ${\mathbf{Z^{\prime}R}}^{{ - {\mathbf{1}}}} {\mathbf{Z}}$ matrix that was calculated using all the sires. This process was replicated 100 times to calculate the confidence interval for the accuracy.

**Fig. 1:**
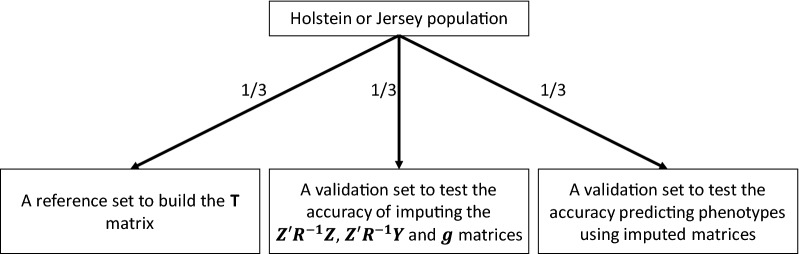
A diagram showing the populations used to test the accuracy of imputing the component matrices for the MetaGS analysis

To test the accuracy of imputing the ${\mathbf{Z^{\prime}R}}^{{ - {\mathbf{1}}}} {\mathbf{Z}}$ matrix and the ${\mathbf{Z^{\prime}R}}^{{ - {\mathbf{1}}}} {\mathbf{y}}$ and ${\mathbf{g}}$ vectors, each of the Holstein or Jersey populations were divided into three equal sets (Fig. 1). One third was randomly selected as a reference to build the ${\mathbf{T}}$ matrix (Eq. (13)). Another third of the remaining bulls was used to validate the imputation accuracy. Masking different proportions (ranging from 0.01 to 0.95 with a 0.05 increment) of the SNPs was randomly applied to the validation bulls to be imputed. The accuracy was inferred from the correlation between the imputed SNPs in the three matrices or vectors (${\mathbf{Z^{\prime}R}}^{{ - {\mathbf{1}}}} {\mathbf{Z}}$, ${\mathbf{Z^{\prime}R}}^{{ - {\mathbf{1}}}} {\mathbf{y}}$ and ${\mathbf{g}}$) and their values in the original three matrices or vectors calculated using all the SNPs. This process was replicated 100 times to calculate the confidence interval for the accuracy. The population used to validate the imputation method was also used as a training population to run the MetaGS model using the imputed matrices (all masking proportions and replicates). The last third of the Holstein and Jersey populations was used to calculate and compare the phenotype prediction accuracies using SNP effects calculated for all SNP masking scenarios using two types of inputs: ${\mathbf{Z^{\prime}R}}^{{ - {\mathbf{1}}}} {\mathbf{Z}}$ plus ${\mathbf{g}}$ or ${\mathbf{Z^{\prime}R}}^{{ - {\mathbf{1}}}} {\mathbf{Z}}$ plus ${\mathbf{Z^{\prime}R}}^{{ - {\mathbf{1}}}} {\mathbf{y}}$. Here, the accuracy was inferred from the correlations between DGV and masked phenotypes.

## Results

**Fig. 2:**
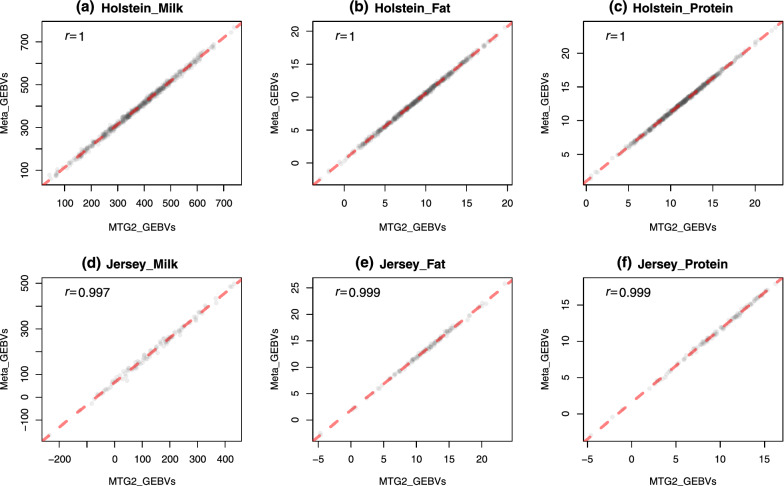
Pearson correlation between genomic estimated breeding values (GEBV) calculated using the MetaGS (y-axis) and mBLUP models using MTG2 (x-axis) for **a** milk yield for Holstein, **b** fat yield for Holstein, **c** protein yield for Holstein, **d** milk yield for Jersey, **e** fat yield for Jersey, and **f** protein yield for Jersey

The meta-analysis developed here (MetaGS) was compared to the single trait (ST) analysis as well as the joint analysis (mBLUP) that exploits raw data for both Australian Holstein (HOL) and Jersey (JER) cattle breeds for three traits. Compared to the ST model, the average correlation across traits between SNP effects produced by MetaGS and ST was 0.97 for HOL and 0.76 for JER (data not shown). MetaGS performed almost the same as mBLUP and they produced highly correlated SNP effects with correlation coefficient values ranging from 0.98 to 0.99. The correlation was even higher (*r* = 1) when comparing the GEBV for all traits in both HOL and JER populations (Fig. 2).

Testing the three models on the validation sets showed comparable prediction accuracies for the three traits when predicting the GEBV of a breed using the SNP effects that were trained on the same reference breed. The average prediction accuracies for both breeds were 0.487, 0.498 and 0.503 for the ST, mBLUP and MetaGS models, respectively (Table 1). However, when performing across-breed prediction (i.e. predicting HOL from JER SNP effects and via versa), the mBLUP and MetaGS models showed superiority compared to the ST model. Predicting the JER validation set using the HOL SNP effects of the ST model had an average prediction accuracy of only 0.043, while the value was equal to 0.397 and 0.387 for the mBLUP and MetaGS models, respectively. Similarly, predicting the HOL validation set using the JER SNP effects had average prediction accuracies of 0.217, 0.403 and 0.42 for the ST, mBLUP and MetaGS models, respectively (Table 1).

**Table 1:** Prediction accuracy for milk, fat and protein yields of sires with phenotyped daughters, using single trait analysis (ST), multi-trait analysis (mBLUP) and meta-analysis (MetaGS)

Trait	Val\Ref	ST		mBLUP		MetaGS	
		JER	HOL	JER	HOL	JER	HOL
Milk	JER	0.52	0.32	0.50	0.49	0.53	0.50
	HOL	0.05	0.51	0.46	0.52	0.46	0.52
Fat	JER	0.34	0.18	0.37	0.36	0.37	0.36
	HOL	0.00	0.52	0.31	0.53	0.30	0.53
Protein	JER	0.55	0.15	0.54	0.39	0.54	0.40
	HOL	0.08	0.48	0.39	0.53	0.40	0.53

The values represent the prediction accuracy (as the correlation coefficient between DGV and the phenotypes of the validation population) for the validation population (rows) using SNP effects calculated on the reference population (columns)

*Jer* Jersey, *Hol* Holstein

**Fig. 3:**
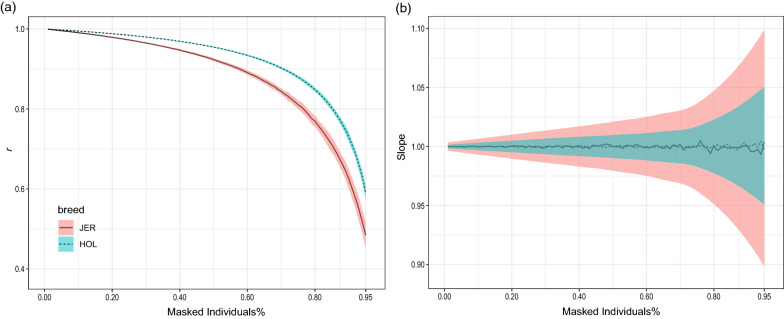
**a** Average correlation coefficients and **b** average regression slope between the original ${\mathbf{Z^{\prime}R}}^{{ - {\mathbf{1}}}} {\mathbf{Z}}$ matrix calculated using the whole Jersey (solid line) and Holstein (dashed line) populations and the rescaled ${\mathbf{Z^{\prime}R}}^{{ - {\mathbf{1}}}} {\mathbf{Z}}$ matrices calculated after masking different proportions of the individuals. Colored ribbons represent the 95% confidence interval of the estimations; red: Jersey and blue: Holstein

Rescaling the ${\mathbf{Z^{\prime}R}}^{{ - {\mathbf{1}}}} {\mathbf{Z}}$ matrix using Eq. (12) has a practical advantage to avoid sharing multiple ${\mathbf{Z^{\prime}R}}^{{ - {\mathbf{1}}}} {\mathbf{Z}}$ matrices if the meta-analysis study was planned for multiple traits measured on different overlapping sets of individuals within the same population. Figure 3a shows the accuracy and standard deviation of rescaling ${\mathbf{Z^{\prime}R}}^{{ - {\mathbf{1}}}} {\mathbf{Z}}$ for the HOL and JER populations when masking a proportion of all individuals (between 1 and 95%). The method preserved a high accuracy (> 0.9 for JER and > 0.95 for HOL) even when more than 50% of the population was masked. Moreover, the method did not magnify or shrink the rescaled ${\mathbf{Z^{\prime}R}}^{{ - {\mathbf{1}}}} {\mathbf{Z}}$ compared to the original one since the regression slope always had an average close to 1 with small 95% confidence intervals of 0.01 for HOL and 0.02 for JER when masking 50% of the population (Fig. 3b).

**Fig. 4:**
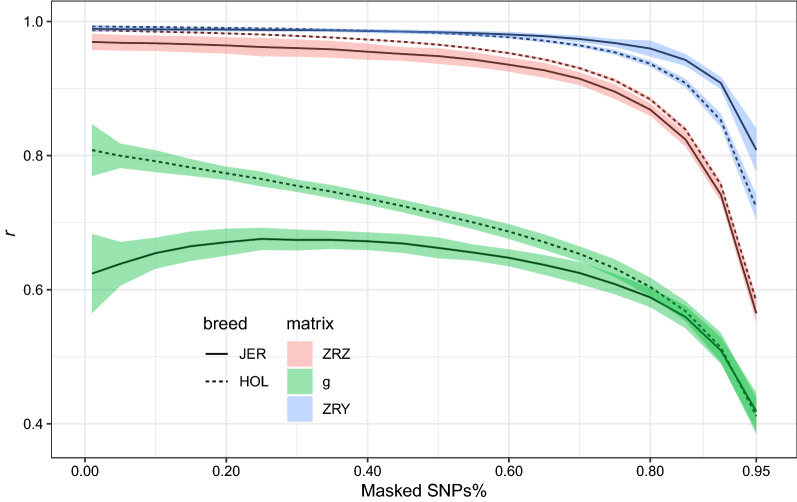
Accuracy of imputing ${\mathbf{Z^{\prime}R}}^{{ - {\mathbf{1}}}} {\mathbf{Z}}$ matrix (red), and ${\mathbf{Z^{\prime}R}}^{{ - {\mathbf{1}}}} {\mathbf{y}}$ (blue) and **g** (green) vectors for both Jersey (solid lines) and Holstein (dashed lines) populations at different SNP masking proportions (x-axis). Colors represent the 95% confidence interval of the estimations

**Fig. 5:**
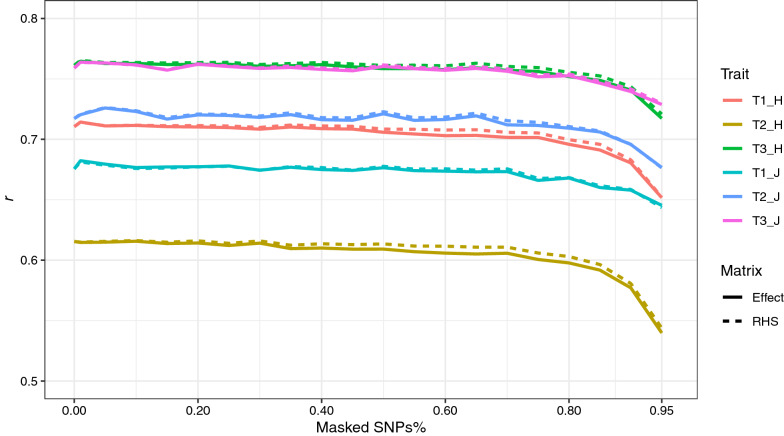
Genomic prediction accuracy using imputed vectors (**g**, solid lines; ${\mathbf{Z^{\prime}R}}^{{ - {\mathbf{1}}}} {\mathbf{y}}$, dashed lines) at different SNP masking proportions (x-axis), based on random cross-validations, for the three traits (T1: milk yield, T2: fat and T3 protein) in both the Holstein (H) and Jersey (J) populations

Different data holders might use different sets of SNPs. Even when different populations were genotyped with the same genotyping platform or chip, they may have filtered them differently. Thus, we proposed a method to impute missing variants in the ${\mathbf{Z^{\prime}R}}^{{ - {\mathbf{1}}}} {\mathbf{Z}}$ matrix and the ${\mathbf{Z^{\prime}R}}^{{ - {\mathbf{1}}}} {\mathbf{y}}$ and ${\mathbf{g}}$ vectors without accessing the raw data using an independent reference population. The accuracy of imputing the ${\mathbf{g}}$ vector was low even when only 1% of the SNPs were masked (0.81 for HOL and 0.63 for JER; Fig. 4). However, the method imputed the ${\mathbf{Z^{\prime}R}}^{{ - {\mathbf{1}}}} {\mathbf{Z}}$ matrix and the ${\mathbf{Z^{\prime}R}}^{{ - {\mathbf{1}}}} {\mathbf{y}}$ vector with high accuracy even at high SNP masking rates. The left-hand side matrix had an accuracy higher than 0.92 when up to 70% of the SNPs were masked while the right-hand side had an accuracy higher than 0.9 when up to 90% of the SNPs were masked in both HOL and JER breeds (Fig. 4). However, the low accuracy of predicting the ${\mathbf{g}}$ vector did not affect the final prediction accuracy results, regardless of whether we used the ${\mathbf{g}}$ vector or the ${\mathbf{Z^{\prime}R}}^{{ - {\mathbf{1}}}} {\mathbf{y}}$ vector in the MetaGS analysis (Fig. 5). Our results clearly demonstrated no changes in DGV prediction accuracy when using imputed vectors (${\mathbf{g}}$ or ${\mathbf{Z^{\prime}R}}^{{ - {\mathbf{1}}}} {\mathbf{y}}$) with a masking proportion of up to 70% compared to the original scenario that used the complete dataset, i.e. the masking proportion is equal to zero for all traits in both Holstein and Jersey populations (Fig. 5).

## Discussion

### Accuracy of MetaGS

We demonstrated that the MetaGS model can duplicate the estimation of SNP effects and prediction results of the mBLUP model. The outcomes of both models were highly correlated in all tested scenarios. For the Holstein population, SNP effects of both models were highly correlated with the ST model (*r* = 0.97) unlike the Jersey population which had an average correlation coefficient of 0.76. This is mainly due to the large reference population for the Holstein breed (4105 bulls) compared to the Jersey breed (1071 bulls), which limited the effect of the Jersey’s input on the Holstein SNP effects. On the other hand, the smaller Jersey population benefits more from its correlation with the larger Holstein population.

Applying SNP effects on the validation population showed that the mBLUP and MetaGS models had no accuracy gain over the ST model when predicting a breed’s performance using the SNP effects of the same breed. This result might be specific to our data as previous studies reported higher accuracy of multi-trait models over single-trait models [16, 21]. Anyway, it is outside the scope of the current paper to demonstrate the advantage of mBLUP or MetaGS over the ST model since MetaGS was developed to reproduce mBLUP results without accessing raw data. However, mBLUP and MetaGS had on average nine times and two times higher accuracy for the Jersey and Holstein breeds, respectively, compared to the ST model when predicting the performance of one breed using the SNP effects of the other breed. This is expected given that mBLUP and MetaGS use the information of different breeds. Different breeds usually have different LD structures [22]. Consequently, 50k SNPs is not a sufficiently dense SNP panel so that the correlation between SNPs and causal variants is the same in Holstein and Jersey.

Unlike other models that produce a global effect value per SNP [14, 16], MetaGS calculates population-specific SNP effects considering variation in LD structures and genetic correlation among populations. For this reason, the model can fit complex traits with high genotype × environment interactions without reducing their accuracies. Even in the most extreme scenario in which different populations were completely uncorrelated, i.e. the genetic correlation coefficients among populations were equal to zero, the resulting SNP effects after running MetaGS will be equal to the input population-specific effects obtained from the ST analysis, assuming no imputation is required.

### Solving practical difficulties

The two major issues that limit the application of meta-analyses to combine SNP effects of different genomic prediction studies are the differences in analyzed variants and the size of the required summary statistic files that need to be shared. The framework of the MetaGS method is flexible enough to solve these issues, which makes it more acceptable from a practical point of view.

#### Rescaling the ${\mathbf{Z^{\prime}R}}^{{ - {\mathbf{1}}}} {\mathbf{Z}}$ matrix

For different measured traits on the same population, usually the number of phenotyped individuals varies slightly, while other traits are measured on a small proportion of the population. For this reason, each trait should have its own ${\mathbf{Z^{\prime}R}}^{{ - {\mathbf{1}}}} {\mathbf{Z}}$ matrix. The dimension of this matrix is $m \times m$, where $m$ is the number of SNPs, making it a huge matrix for sharing, especially if the meta-analysis was planned for multiple traits. Our results showed that there is no need to share multiple ${\mathbf{Z^{\prime}R}}^{{ - {\mathbf{1}}}} {\mathbf{Z}}$ matrices for the same population as these can be accurately predicted from one to another using Eq. (12) even when more than half of the population had no phenotypic records. The ${\mathbf{Z^{\prime}R}}^{{ - {\mathbf{1}}}} {\mathbf{Z}}$ matrix represents the LD structure in the population, which makes it redundant for different traits except for a difference in scale that is inferred from the number of phenotyped individuals. For this reason, it is recommended that each data holder should calculate a single ${\mathbf{Z^{\prime}R}}^{{ - {\mathbf{1}}}} {\mathbf{Z}}$ matrix using all the genotyped individuals even if they do not have phenotypes and this can be rescaled for each trait depending on the number of individuals having phenotypic records. However, sharing ${\mathbf{Z^{\prime}R}}^{{ - {\mathbf{1}}}} {\mathbf{Z}}$ matrices may still be required for traits with very small reference populations or deviated ${\mathbf{R}}^{{ - {\mathbf{1}}}}$ patterns. As this matrix is symmetric, data holders need to share only the upper (or lower) triangle with the diagonal. This information can be saved in files with binary format to further reduce the size of the transferred materials.

#### Imputing summary statistics for missing variants

MetaGS provided a comprehensive mathematical frame to synchronize variants in different datasets by imputing them from a reference population without accessing the raw data using the ${\mathbf{T}}$ matrix. We showed that our imputation method had a minimal effect on prediction accuracy even at the high SNP masking rate of 70%. Calculating the ${\mathbf{T}}$ matrix requires inverting the ${\mathbf{Z^{\prime}}}_{i} {\mathbf{Z}}_{i}$ matrix and in order to make it invertible, the number of variants must be smaller than the number of individuals in the reference population. While it is impossible to get such a large reference population for most organisms, we recommend applying Eq. (13) within each LD block, separately, and setting all off-diagonal or inter LD block elements to zero. In our analysis, we calculated the ${\mathbf{T}}$ matrix for each 200 adjacent SNPs, but the accuracy was not affected when using different numbers of SNPs per LD block (data not shown).

In genomic prediction, the effect of a causal variant can be distributed over multiple variants in high LD with it since they are all fitted together in one model [23], unlike in GWAS in which overlapping sources of variation can be attributed to multiple variants fitted independently [5]. For this reason, unsynchronized variants can be easily filtered out in GWAS as they were fitted independently but this cannot be done in genomic prediction. This can explain why the accuracy of imputing the ${\mathbf{g}}$ vector was relatively low compared to that of the ${\mathbf{Z^{\prime}R}}^{{ - {\mathbf{1}}}} {\mathbf{Z}}$ matrix and ${\mathbf{Z^{\prime}R}}^{{ - {\mathbf{1}}}} {\mathbf{y}}$ vector even at very low SNP masking rates such as 1% (Fig. 4). Different variants in high LD can have variable effect values but they would end with comparable values on the right-hand side (or the ${\mathbf{Z^{\prime}R}}^{{ - {\mathbf{1}}}} {\mathbf{y}}$ vector) when they are multiplied with the allelic dosage for each individual or the ${\mathbf{Z^{\prime}R}}^{{ - {\mathbf{1}}}} {\mathbf{Z}}$ matrix. This can explain why the prediction accuracy did not change when using the ${\mathbf{Z^{\prime}R}}^{{ - {\mathbf{1}}}} {\mathbf{y}}$ vector compared to the analysis that used the ${\mathbf{g}}$ vector as input (Fig. 5). It is worth noting that the accuracies in Fig. 5 are much larger than those in Table 1 given that in the former, one random third of the population was used for validation, while in the latter, young bulls that were born after 2010 were used for validation. Therefore, the reference and validation populations in the former were more related.

While our results demonstrated that missing variants can be imputed with high accuracy even at high variant masking rates, it is worth noting that the imputation accuracy depends on the relatedness between the tested population and the reference populations, like any other imputation method [24]. The reference population assumed here is expected to be genotyped with all the variants used across studies. One option to collect such a population, if it is not available, is to select a subset of representative individuals within each population to be genotyped with the full list of variants that are planned to be used in the meta-analysis. These individuals can be used within each population to impute other individuals with any imputation algorithm such as FImpute [24] or Minimac [25]. Another option is to have an agreement among all data holders to share the genotyping of a few random individuals (e.g. 100 or 200 individuals) without sharing their ID or phenotypes. Participants can share a single random phased haplotype per individual so the actual genotype cannot be revealed, which allows them to share large numbers of haplotypes without any risk. Any imputation algorithm can then be applied after gathering all the individuals to fill the missing variants across populations. The imputed data can then be returned to the data holders to be used to impute the rest of the population. Sharing such information cannot be used for any purpose except for building the ${\mathbf{T}}$ matrix in Eq. (13).

## Conclusions

We developed a meta-genomic prediction method (MetaGS) that accurately duplicated the results of the standard multi-trait best linear unbiased prediction (mBLUP) method without directly analyzing the raw data. Highly correlated SNP effects (*r* > 0.98) and almost the exact genomic estimated breeding values (*r* > 0.997) were obtained when applying both MetaGS and mBLUP on fat, protein and milk yields of Australian Holstein and Jersey data. The method was extended to synchronize the variants among different populations using a shared reference population. This was achieved by imputing missing variants from the shared summary statistics without the need to apply imputation on the individual-level data. We also developed a method to facilitate the sharing of summary statistics to avoid sharing multiple large files for different traits. MetaGS is not restricted to bull’s data and can be applied to any organism and can consider the variation in genetic correlation between participant populations. For example, in plant breeding, e.g., wheat, different breeding companies targeting distant environments belonging to the same mega environment could benefit from sharing their results within the analytical framework of MetaGS [26]. However, for the evaluation of bulls, the current meta-analyses MACE and GMACE must continue to provide the essential service of comparing foreign to domestic bulls. Otherwise, genetic progress may be much slower if breeders use only local animals instead of using the better foreign animals.

## Data Availability

DataGene (DataGene Ltd., Melbourne, Australia; https://datagene.com.au/) are the custodians of the raw phenotype and genotype data of Australian dairy cows. Research-related requests for access to the data may be accommodated on a case-by-case basis.

## References

[1] Meuwissen THE, Hayes BJ, Goddard ME (2001). Prediction of total genetic value using genome-wide dense marker maps. Genetics.

[2] Crossa J, Perez P, Hickey J, Burgueno J, Ornella L, Cerón-Rojas J (2014). Genomic prediction in CIMMYT maize and wheat breeding programs. Heredity (Edinb).

[3] Goddard ME, Hayes BJ, Meuwissen TH (2011). Using the genomic relationship matrix to predict the accuracy of genomic selection. J Anim Breed Genet.

[4] Abraham G, Inouye M (2015). Genomic risk prediction of complex human disease and its clinical application. Curr Opin Genet Dev.

[5] Wray NR, Lee SH, Mehta D, Vinkhuyzen AA, Dudbridge F, Middeldorp CM (2014). Research review: polygenic methods and their application to psychiatric traits. J Child Psychol Psychiatry.

[6] Hayes BJ, Bowman PJ, Chamberlain AJ, Goddard ME (2009). Invited review: genomic selection in dairy cattle: progress and challenges. J Dairy Sci.

[7] Tenopir C, Allard S, Douglass K, Aydinoglu AU, Wu L, Read E (2011). Data sharing by scientists: practices and perceptions. PLoS One.

[8] Evangelou E, Ioannidis JP (2013). Meta-analysis methods for genome-wide association studies and beyond. Nat Rev Genet.

[9] Glass GV (1976). Primary, secondary, and meta-analysis of research. Educ Res.

[10] Schaeffer LR (1994). Multiple-country comparison of dairy sires. J Dairy Sci.

[11] VanRaden PM, Sullivan PG (2010). International genomic evaluation methods for dairy cattle. Genet Sel Evol.

[12] Sullivan PG, Zumbach B, Durr JW, Jakobsen JH (2011). International genomic evaluations for young bulls. Interbull Bull.

[13] Bolormaa S, Pryce JE, Reverter A, Zhang Y, Barendse W, Kemper K (2014). A multi-trait, meta-analysis for detecting pleiotropic polymorphisms for stature, fatness and reproduction in beef cattle. PLoS Genet.

[14] Maier RM, Zhu Z, Lee SH, Trzaskowski M, Ruderfer DM, Stahl EA (2018). Improving genetic prediction by leveraging genetic correlations among human diseases and traits. Nat Commun.

[15] Pasaniuc B, Price AL (2017). Dissecting the genetics of complex traits using summary association statistics. Nat Rev Genet.

[16] Vandenplas J, Calus MP, Gorjanc G (2018). Genomic prediction using individual-level data and summary statistics from multiple populations. Genetics.

[17] Liu Z, Goddard ME, Hayes BJ, Reinhardt F, Reents R (2016). Technical note: equivalent genomic models with a residual polygenic effect. J Dairy Sci.

[18] VanRaden PM (2008). Efficient methods to compute genomic predictions. J Dairy Sci.

[19] Strandén I, Christensen OF (2011). Allele coding in genomic evaluation. Genet Sel Evol.

[20] Lee SH, van der Werf JH (2016). MTG2: an efficient algorithm for multivariate linear mixed model analysis based on genomic information. Bioinformatics.

[21] Raymond B, Bouwman AC, Wientjes YC, Schrooten C, Houwing-Duistermaat J, Veerkamp RF (2018). Genomic prediction for numerically small breeds, using models with pre-selected and differentially weighted markers. Genet Sel Evol.

[22] de Roos APW, Hayes BJ, Spelman RJ, Goddard ME (2008). Linkage disequilibrium and persistence of phase in Holstein-Friesian, Jersey and Angus cattle. Genetics.

[23] Daetwyler HD, Villanueva B, Woolliams JA (2008). Accuracy of predicting the genetic risk of disease using a genome-wide approach. PLoS One.

[24] Sargolzaei M, Chesnais JP, Schenkel FS (2014). A new approach for efficient genotype imputation using information from relatives. BMC Genomics.

[25] Das S, Forer L, Schönherr S, Sidore C, Locke AE, Kwong A (2016). Next-generation genotype imputation service and methods. Nat Genet.

[26] Jighly A, Hayden M, Daetwyler H (2021). Integrating genomic selection with a genotype plus genotype x environment (GGE) model improves prediction accuracy and computational efficiency. Plant Cell Environ.

